# Supplementary material for: One-sided device-independent random number generation through fiber channels
Source: Light Sci Appl. 2025 Jan 3;14:25. doi: 10.1038/s41377-024-01641-9 (PMC11696713; doi:10.1038/s41377-024-01641-9)
Supplement: Supplementary file 1 — Supplementary Information for “One-Sided Device-Independent Random Number Generation Through Fiber Channels” [file 41377_2024_1641_MOESM1_ESM.pdf]

Supplementary Information for "One-Sided Device-Independent Random Number Generation Through Fiber Channels"

Jinfang Zhang,[1, *] Yi Li,[2, 3, *] Mengyu Zhao,[1] Dongmei Han,[1] Jun Liu,[1] Meihong Wang,[1,4] Qihuang Gong,[2,4,5] Yu Xiang,[2,4,†] Qiongyi He,[2,4,5,6] and Xiaolong Su[1,4,‡]

[1] State Key Laboratory of Quantum Optics and Quantum Optics Devices, Institute of Opto-Electronics, Shanxi University, Taiyuan, 030006, China

[2] State Key Laboratory for Mesoscopic Physics, School of Physics, Frontiers Science Center for Nano-optoelectronics, & Collaborative Innovation Center of Quantum Matter, Peking University, Beijing 100871, China

[3] Beijing Academy of Quantum Information Sciences, Beijing 100193, China

[4] Collaborative Innovation Center of Extreme Optics, Shanxi University, Taiyuan, Shanxi 030006, China

[5] Peking University Yangtze Delta Institute of Optoelectronics, Nantong, Jiangsu 226010, China

[6] Hefei National Laboratory, Hefei 230088, China

DENSITY MATRIX OF TWO-MODE GAUSSIAN STATE FROM ITS WIGNER FUNCTION

For an experimental CM,

$$\sigma = \begin{bmatrix} \sigma_{11} & 0 & \sigma_{13} & 0 \\ 0 & \sigma_{22} & 0 & \sigma_{24} \\ \sigma_{31} & 0 & \sigma_{33} & 0 \\ 0 & \sigma_{42} & 0 & \sigma_{44} \end{bmatrix} \tag{1}$$

its inverse σ^{-1} can be written as

$$\sigma^{-1} = \begin{bmatrix} a & 0 & e & 0 \\ 0 & c & 0 & f \\ e & 0 & b & 0 \\ 0 & f & 0 & d \end{bmatrix} \tag{2}$$

Within the Gaussian state assumption, the corresponding multivariate Wigner function is [1]

$$W_\rho(\xi) = \frac{1}{(2\pi)^2\sqrt{\det\sigma}} \exp[-(1/2)(\xi-\delta)^T \sigma^{-1}(\xi-\delta)] \tag{3}$$

where $\xi \in \mathbb{R}^4$ denotes a phase-space coordinate vector, and $\delta = \langle \hat{R} \rangle$ is the displacement vector. In this experiment, $\delta = 0$. The elements of the corresponding two-mode Gaussian state can be obtained from its Wigner function $W_\rho(q_A, q_B, p_A, p_B)$:

$$\langle nk|\rho|ml\rangle = 16\pi^2 \iiiint_{\mathbb{R}} W_\rho(q_A,q_B,p_A,p_B) W_{|n\rangle\langle m|}(q_A,p_A) W_{|k\rangle\langle l|}(q_B,p_B)\, dq_A dq_B dp_A dp_B. \tag{4}$$

Here the Wigner function of each matrix element is

$$W_{|n\rangle\langle m|}(q,p) = \frac{(-1)^n}{2\pi}\left(\frac{n!}{m!}\right)^{1/2} e^{-(q^2+p^2)/2}(q+ip)^{m-n}\mathcal{L}_n^{m-n}(q^2+p^2) \tag{5}$$

where $\mathcal{L}_n^{\alpha}(x)$ is a generalized Laguerre polynomials. In the following, we will take its closed formula [2] $\mathcal{L}_n^{\alpha}(x) = \sum_{m=0}^{n} (-1)^m \binom{n+\alpha}{n-m} \frac{x^m}{m!}$. However, numerically reconstructing the density matrix of a two-mode state from its Wigner function is computationally demanding, especially when we cut off it at a high Fock basis. It is then desirable to analytically derive the density matrix of the two-mode Gaussian state from the experimental CM. We analytically give the elements of this density matrix $(m \ge n,\ l \ge k)$:

$$\langle nk|\rho|ml\rangle = C_{nkml} \sum_{t=0}^{m-n}\sum_{s=0}^{l-k}\sum_{j=0}^{n}\sum_{u=0}^{j}\sum_{q=0}^{k}\sum_{h=0}^{q} D_{tsjuqh}^{nkml} f(g_{q_A}, g_{q_B}, a, b, e) f(g_{p_A}, g_{p_B}, c, d, f) \tag{6}$$

where $g_{q_A} = t + 2u$, $g_{q_B} = s + 2h$, $g_{p_A} = m - n - t + 2(j-u)$, $g_{p_B} = l - k - s + 2(q-h)$, and

$$
\begin{aligned}
C_{nkml} &= \frac{16(-1)^{n+k}}{\pi^2\sqrt{\det\sigma}}\sqrt{\frac{n!k!}{m!l!}}\,2^{(m-n+l-k)/2} \\
D^{nkml}_{tsjuqh} &= \binom{m-n}{t}\binom{l-k}{s}\binom{m}{n-j}\binom{j}{u}\binom{l}{k-q}\binom{q}{h}(-1)^{j+q}\frac{2^{q+j}}{j!q!}i^{m-n-t+l-k-s} \\
f(\mathcal{G}_A,\mathcal{G}_B,\mathcal{A},\mathcal{B},\mathcal{E}) &= \sum_{v=0}^{\mathcal{G}_A}\binom{\mathcal{G}_A}{v}\left(\frac{-\mathcal{E}}{\mathcal{A}+1}\right)^{\mathcal{G}_A-v}\frac{\Gamma\left(\frac{v+1}{2}\right)\Gamma\left(\frac{\mathcal{G}_B+\mathcal{G}_A-v+1}{2}\right)\delta_{\mathcal{G}_B+\mathcal{G}_A-v,even}\delta_{v,even}}{(\mathcal{A}+1)^{\frac{v+1}{2}}\left(\mathcal{B}+1-\frac{\mathcal{E}^2}{\mathcal{A}+1}\right)^{\frac{\mathcal{G}_B+\mathcal{G}_A-v+1}{2}}}
\end{aligned}
\tag{7}
$$

where $\Gamma(z)=\int_0^\infty t^{z-1}e^{-t}dt$, and $\Gamma(n+\frac{1}{2})=\binom{n-\frac{1}{2}}{n}n!\sqrt{\pi}$ for non-negative integer values of n . For $m \ge n, l < k$, the expression of $\langle nk|\rho|ml\rangle$ holds the same function with $\langle nl|\rho|mk\rangle$ except the coefficient D^{nlmk}_{tsjuqh} multiplies with $(-1)^{k-l-s}$.

The derivation of Eq. (6) is given in the following. Here the density matrix of its corresponding two-mode Gaussian state can be expressed by the parameters in Eq. (2):

$$
\begin{aligned}
\langle nk|\rho|ml\rangle = C_{nkml}&\sum_{t=0}^{m-n}\sum_{s=0}^{l-k}\sum_{j=0}^{n}\sum_{u=0}^{j}\sum_{q=0}^{k}\sum_{h=0}^{q}\binom{m-n}{t}\binom{l-k}{s}\binom{m}{n-j}\binom{j}{u}\binom{l}{k-q}\binom{q}{h}(-1)^{j+q}\frac{2^{q+j}}{j!q!}i^{m-n-t+l-k-s} \\
&\times\iint_{\mathbb{R}}\left\{\exp\left[-(q_A^2+q_B^2)-aq_A^2-2eq_Aq_B-bq_B^2\right]q_A^{t+2u}q_B^{s+2h}\right\}dq_Adq_B \\
&\times\iint_{\mathbb{R}}\left\{\exp\left[-(p_A^2+p_B^2)-cp_A^2-2fp_Ap_B-dp_B^2\right]p_A^{m-n-t+2(j-u)}p_B^{l-k-s+2(q-h)}\right\}dp_Adp_B
\end{aligned}
\tag{8}
$$

Here the first integral of q_A and q_B is

$$
\begin{aligned}
&\iint_{\mathbb{R}}\left\{\exp\left[-(q_A^2+q_B^2)-aq_A^2-2eq_Aq_B-bq_B^2\right]q_A^{g_{qA}}q_B^{g_{qB}}\right\}dq_Adq_B \\
&=\sum_{v=0}^{g_{qA}}\binom{g_{q_A}}{v}\delta_{v,even}\frac{\Gamma\left(\frac{v+1}{2}\right)}{(a+1)^{(v+1)/2}}\int_{\mathbb{R}}\left(\frac{-e}{a+1}\right)^{g_{qA}-v}\exp\left[-\left(b+1-\frac{e^2}{a+1}\right)q_B^2\right]q_B^{g_{qB}+g_{qA}-v}dq_B \\
&=\sum_{v=0}^{g_{qA}}\binom{g_{q_A}}{v}\left(\frac{-e}{a+1}\right)^{g_{qA}-v}\frac{\Gamma\left(\frac{v+1}{2}\right)\Gamma\left(\frac{g_{q_B}+g_{q_A}-v+1}{2}\right)}{(a+1)^{\frac{v+1}{2}}\left(b+1-\frac{e^2}{a+1}\right)^{\frac{g_{qB}+g_{qA}-v+1}{2}}}\delta_{v,even}\delta_{g_{qB}+g_{qA}-v,even}
\end{aligned}
\tag{9}
$$

Similarly, the second integral is

$$
\begin{aligned}
&\iint_{\mathbb{R}}\left\{\exp\left[-(p_A^2+p_B^2)-cp_A^2-2fp_Ap_B-dp_B^2\right]p_A^{g_{pA}}p_B^{g_{pB}}\right\}dp_Adp_B \\
&=\sum_{y=0}^{g_{pA}}\binom{g_{p_A}}{y}\left(\frac{-f}{c+1}\right)^{g_{pA}-y}\frac{\Gamma\left(\frac{y+1}{2}\right)\Gamma\left(\frac{g_{p_B}+g_{p_A}-y+1}{2}\right)}{(c+1)^{\frac{y+1}{2}}\left(d+1-\frac{f^2}{c+1}\right)^{\frac{g_{pB}+g_{pA}-y+1}{2}}}\delta_{y,even}\delta_{g_{pB}+g_{pA}-y,even}
\end{aligned}
\tag{10}
$$

Putting all things together, we give Eq. (6).

However, due to the existence of inevitable noise in the experiment. The density matrix ρ obtained directly from the experimental data is probably not a positive semi-define matrix. Here we reconstruct a physical density matrix that approximates the experimental data:

$$\begin{aligned} &\min_{\rho^{rec}} \quad \frac{1}{2}\| \rho^{rec} - \rho \|_1 \\ &\text{s.t.} \quad \rho^{rec} \geq 0, \ \mathrm{Tr}(\rho^{rec}) = 1 \end{aligned} \tag{11}$$

Here we use the trace norm to denote the distance between ρ^{rec} and ρ, where $\| A\|_1 = \sum_i |\lambda_i|$, and λ_i is the eigenvalue of matrix A. It can be converted to a Semidefinite Programming (SDP):

$$\begin{aligned} &\min_{\rho^{rec}, X} \quad \frac{1}{2}\mathrm{Tr}(X) \\ &\text{s.t.} \quad -X \leq \rho^{rec} - \rho \leq X \\ &\qquad \rho^{rec} \geq 0, \ \mathrm{Tr}(\rho^{rec}) = 1 \end{aligned} \tag{12}$$

By utilizing the reconstructed state ρ^{rec}, we can obtain the assemblage $\sigma_{b|y}^{\text{obs}} = \mathrm{Tr}_B[I \otimes M_{b|y}\rho^{rec}]$ as well as the joint probability $p^{\text{obs}}(ab \mid xy) = \mathrm{Tr}[M_{a|x} \otimes M_{b|y}\rho^{rec}]$ within each coarse-grained protocol. We then optimize all the coarse-graining protocols $\left(T_{\hat{p}} = T_{\hat{q}}\right)$ to certify randomness. Hence, we take the optimal protocol to perform measurements and extract random numbers.

PARAMETERS OF SQUEEZED STATE

The squeezing levels of generated states are mainly limited by the normalized pump power, the total detection efficiency, and the escape efficiency. More specifically, the anticipated variances of the squeezing $\left(\Delta^2 \hat{X}_-\right)$ and anti-squeezing $\left(\Delta^2 \hat{X}_+\right)$ quadratures generated by the OPA can be modeled by:

$$\Delta^2 \hat{X}_{\pm} = 1 \pm \alpha\rho \frac{4x}{\left(1 \mp x\right)^2 + 4\Omega^2} \tag{13}$$

Here, the total detection efficiency $\alpha = \zeta\eta\xi^2\varepsilon$ is determined by the propagation efficiency ζ, the photodiode quantum efficiency η, the homodyne efficiency ξ^2 (ξ is the visibility between the output and the local oscillator modes), and the clearance of the detector ε (the electronic noise is 15 dB below the vacuum noise in our experiment). The normalized pump power is $x = \sqrt{P/P_{th}}$, where P is the second-harmonic pump power, P_{th} is the pump power required to reach the OPA threshold, ρ is the escape efficiency defined as $\rho = T/\left(T + L\right)$, where T and L are the transmission of the output coupler mirror and the intracavity loss, respectively. Ω is the normalized frequency $\Omega = 2\pi f/\gamma$, where f is the measurement frequency, and $\gamma = c\left(T + L\right)/l$ with the speed of light c and the cavity round-trip length l. In our current setup, $\xi = 0.99, \zeta = 0.93, \eta = 0.99, \varepsilon = 0.968, T = 0.125, L = 0.009, \rho = 0.93, l = 0.214\ \text{m}$, and the measurement frequency $f = 4\ \text{MHz}$, therefore, the total detection efficiency $\alpha = 0.87$.

In general, if the total detection efficiency increases, the squeezing levels and the purity of the corresponding squeezed states will increase. What's more, we generated a quadrature phase squeezed state at 4 MHz, which can effectively avoid the influence of technical noise below 2 MHz.

RESULTS OF QRNG AT DIFFERENT TRANSMISSION DISTANCES

In the extraction of random numbers, for a long raw data sequence, we cut it into several blocks, each containing n raw bits. Then, each block is processed using a Toeplitz matrix to generate extracted random bits of length m [3]. The $m \times n$ Toeplitz matrix is in the following form, with the elements being the same in every diagonal.

$$T_{m \times n} = \begin{pmatrix} a_n & a_{n-1} & \cdots & a_2 & a_1 \\ a_{n+1} & a_n & \ddots & & a_2 \\ a_{n+2} & a_{n+1} & \ddots & \ddots & \vdots \\ \vdots & \vdots & & \ddots & a_{m-1} \\ a_{n+m-1} & a_{n+m-2} & \cdots & a_{m+1} & a_m \end{pmatrix} \tag{14}$$

The n-bit-long raw data is

$$R_m = \begin{pmatrix} r_1 \\ r_2 \\ r_3 \\ \vdots \\ r_n \end{pmatrix} \tag{15}$$

The extracted random bits are

$$V_n = \begin{pmatrix} v_1 \\ v_2 \\ v_3 \\ \vdots \\ v_n \end{pmatrix} \tag{16}$$

All extracted random bit segments are then concatenated to produce a final output sequence.

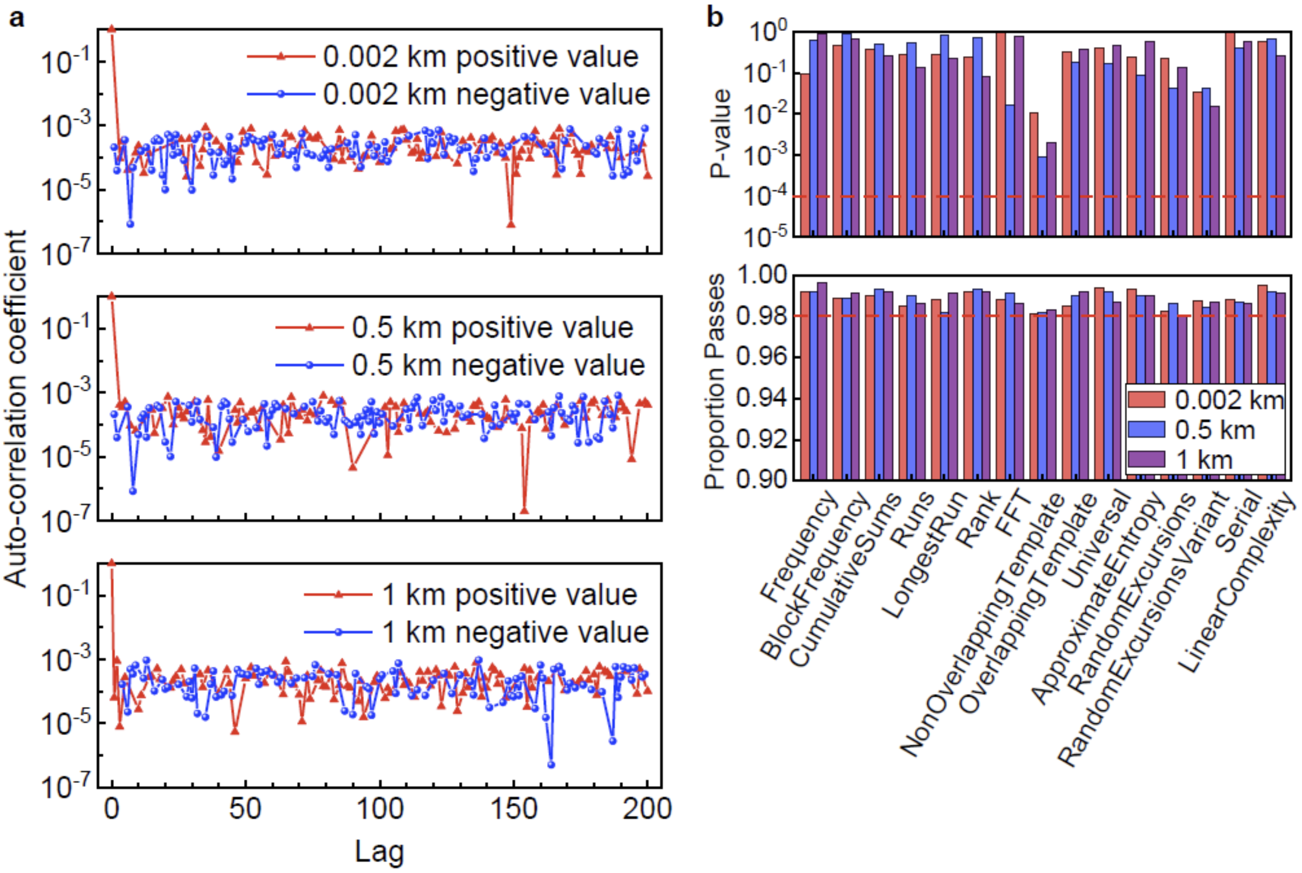

Fig. S1. Results of extracted random numbers at 0.002, 0.5 and 1 km fiber channels. **a** An autocorrelation calculations for random bits. **b** Results of NIST test suite for the random bits.

When the transmission distance is 0.002 km, the $H_{\min}$ of the raw data is about 0.11113 bits per sample. We obtain the random numbers with generation rate of 11.113 Mbits s^{-1} by setting $m = 1024$ and $n = 46{,}100$ for constructing the Toeplitz matrix. As the fiber length increases to 0.5 km, the obtained $H_{\min}$ is 0.105 bits, which leads to a generation rate of 10.500 Mbits s^{-1} with

$m = 1024$ and $n = 48{,}800$. For the 1 km fiber channel between the server and Bob, we obtain $H_{\min} = 0.09253$ bits , which leads to a generation rate of 9.253 Mbits s^{-1} with $m = 1024$ and $n = 55{,}400$.

The autocorrelation analysis is also performed for random bits at these transmission distances. In Fig. S1a, the autocorrelation analysis of extracted bits at transmission distances of 0.002, 0.5, and 1 km are presented from top to bottom, respectively. The corresponding average values of autocorrelation are 2.30×10^{-6}, 3.22×10^{-6} , and 1.74×10^{-5} respectively. The results of the NIST test suite for the obtained random numbers at these transmission distances are shown in Fig. S1b. All the P-values exceed 0.0001, indicating that the sequences conform to a uniform distribution. The corresponding proportions of the sequences that pass the test beyond 0.9805607.

THEORETICAL PREDICTION ON THE TRANSMISSION DISTANCE

To quantify the required squeezing level to extend the transmission distance, we now consider an ideal scenario where a two-mode squeezed state is generated by combining two pure squeezed states with squeezing level s dB. Since the steering-based randomness cannot be certified if Bob cannot steer Alice, by taking the error bar into account, we consider the minimum required initial squeezing such that the remaining steerability $\mathcal{G}^{B\to A} \geq 0.02$ after L km of transmission. As shown in the following Fig. S2, to generate nonzero randomness over a distance of 12 km, one needs to prepare a pure squeezed state with squeezing level of at least ±12 dB (the remaining $H_{\min} = 0.00407$ bits).

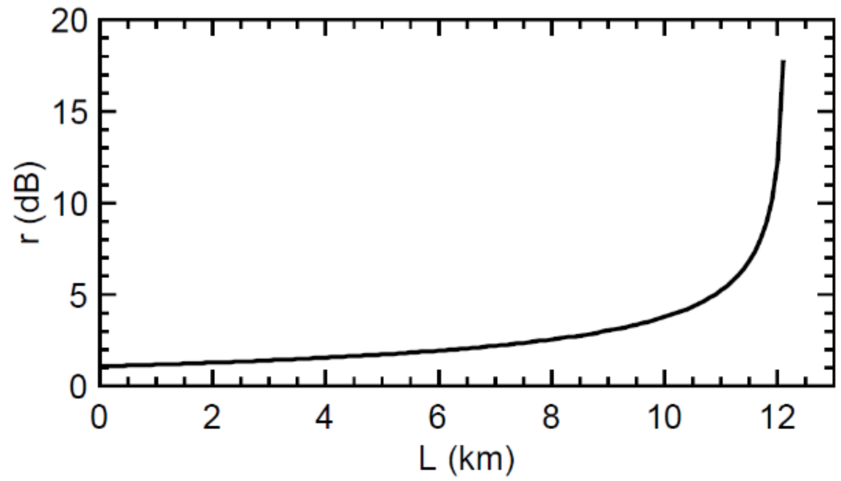

Fig. S2. The minimum required squeezing versus transmission distance (for $\mathcal{G}^{B\to A} = 0.02$)

THE EFFECT OF EXPERIMENTAL PARAMETERS ON THE CERTIFIABLE RANDOMNESS

To provide an estimation on how the certifiable randomness influenced by the imperfection of experimental, we consider the effects of detector loss, strength of the squeezing, and input power on the generated randomness.

We first consider the detection loss, the squeezing values shown in the main text are obtained by directly measuring the state generated by OPA via the imperfect detectors with detection efficiency 87% in our experiment. If the detection efficiency is 100% , OPA1 would generate a squeezed state with squeezing/antisqueezing values of −3.40 dB/+3.81 dB, and OPA2 would have a squeezed state with squeezing/antisqueezing values of −3.29 dB/+3.81 dB. After mixing them on a perfect balanced beam splitter, Bob’s state can be transferred nearly a distance of 9 km such that it can still be used to certify nonzero randomness ($H_{\min} = 1.46\times10^{-4}$ bits for our scheme), as shown in Fig. S3a.

We then consider another ideal scenario where a two-mode entangled state is generated by mixing two pure squeezed states with squeezing level s dB (but one is amplitude-squeezed, the other one is phase-squeezed). At the same time, there is no loss in the optical fiber and detectors. As shown in Fig. S3b, the certified min-entropy $H_{\min}$ increases with strength of the pure

squeezing.

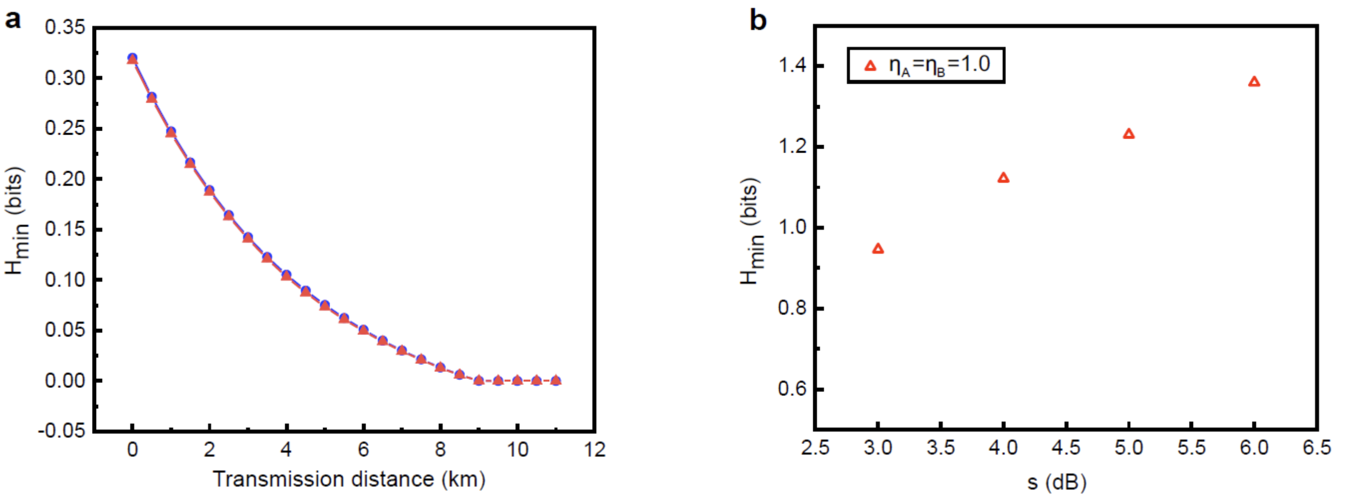

Fig. S3. **a** The certifiable randomness versus the transmission distance with the perfect detectors and beam splitters. **b** The certifiable randomness versus strength of the pure squeezing.

Finally, with the detection efficiency at 0.87 and the beam splitter's splitting ratio set at 50:50, the Gaussian steerabilities $\mathcal{G}^{B\to A}$ and min-entropy $H_{\min}$ against the input pump power are plotted in Fig. S4a and S4b. As the pump power increases, the change in Gaussian steerabilities $\mathcal{G}^{B\to A}$ and min-entropy $H_{\min}$ tends to level off, and min-entropy even decreases after reaching 120 mW.

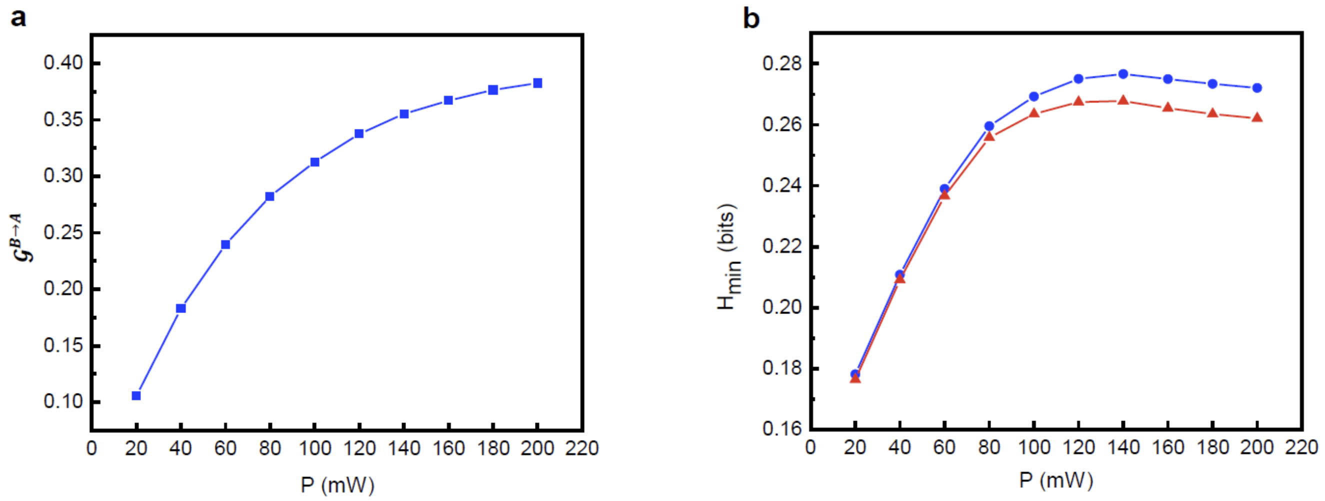

Fig. S4**. a** The Gaussian steerabilities $\mathcal{G}^{B\to A}$ and **b** the certified randomness $H_{\min}$ based on the conditional state (blue) or the joint probabilities (red) versus input pump power.

So, we can improve the strength of the squeezing while maintaining the purity of the squeezed state, increase the detector efficiency, reduce coupling loss, and balance the beam splitter's splitting ratio to enhance the min-entropy $H_{\min}$ and increase the maximum distance at which QRNG can be reliably performed.

∗ These authors contributed equally

† xiangy.phy@pku.edu.cn

‡ suxl@sxu.edu.cn

Reference

1. Weedbrook, C. et al. Gaussian quantum information. *Reviews of Modern Physics* **84**, 621–669 (2012).

2. Magnus, W. et al. *Formulas and Theorems for the Special Functions of Mathematical Physics* (Springer Berlin, Heidelberg, 1966).

3. Zhao, Z. H., Ma, X. F. & Zhou, H. Y. Performance optimization on practical quantum random number generators: modification on min-entropy evaluation and acceleration on post processing. Print at https://doi.org/10.48550/arXiv.2011.04130 (2020).
